# Supplementation of the Combination of Quercetin and Vitamin E Alleviates the Effects of Heat Stress on the Uterine Function and Hormone Synthesis in Laying Hens

**DOI:** 10.3390/ani14111554

**Published:** 2024-05-24

**Authors:** Xueqing Cao, Felix Kwame Amevor, Xiaxia Du, Youhao Wu, Dan Xu, Shuo Wei, Gang Shu, Jing Feng, Xiaoling Zhao

**Affiliations:** 1 State Key Laboratory of Swine and Poultry Breeding Industry, College of Animal Science and Technology, Sichuan Agricultural University, Chengdu 611130, China; 13835890389@163.com (X.C.); amevorfelix@gmail.com (F.K.A.); 15680811065@163.com (X.D.); wuyouhao2021302122@163.com (Y.W.); 13086605395@163.com (D.X.); 13541399198@163.com (S.W.); 2 Key Laboratory of Livestock and Poultry Multi-omics, Ministry of Agriculture and Rural Affairs, Sichuan Agricultural University, Chengdu 611130, China; 3 Farm Animal Genetic Resources Exploration and Innovation Key Laboratory of Sichuan Province, Sichuan Agricultural University, Chengdu 611130, China; 4 Department of Basic Veterinary Medicine, Sichuan Agricultural University, Chengdu 611130, China; dyysg2005@sicau.edu.cn; 5 Institute of Animal Husbandry and Veterinary Medicine, Tibet Academy of Agricultural and Animal Husbandry Science, Lhasa 851418, China; fengjing0835@sina.com

**Keywords:** calcium metabolism, heat stress, uterus, hormone synthesis, quercetin and vitamin E

## Abstract

**Simple Summary:**

Heat stress usually affects almost all animals, including chickens. However, chickens are more sensitive to heat stress because their capacity to dissipate body heat is low. Hence, in chickens, excessive ambient temperature negatively influences their physiological performance and immunity. In this study, we evaluated the effects of the supplementation of dietary quercetin and vitamin E on the uterine function, eggshell quality via estrogen concentration, calcium metabolism, and antioxidant status of the uterus of laying hens under heat stress. The results show that supplementing the combination of dietary quercetin and vitamin E alleviated the effects of heat stress and improved calcium metabolism, hormone synthesis, and uterine function in the heat-stressed laying hens.

**Abstract:**

Chickens are sensitive to heat stress because their capacity to dissipate body heat is low. Hence, in chickens, excessive ambient temperature negatively influences their reproductive performance and health. Heat stress induces inflammation and oxidative stress, thereby rendering many reproductive organs dysfunctional. In this study, we evaluated the effects of the supplementation of dietary quercetin and vitamin E on the uterine function, eggshell quality via estrogen concentration, calcium metabolism, and antioxidant status of the uterus of laying hens under heat stress. The ambient temperature transformation was set at 34 ± 2 °C for 8 h/d (9:00 am–5:00 pm), which was followed by 22 °C to 28 °C for 16 h/d. Throughout the experiment, the relative humidity in the chicken’s pen was at 50 to 65%. A total of 400 Tianfu breeder hens (120-days-old) were randomly divided into four dietary experimental groups, including basal diet (Control); basal diet + 0.4 g/kg quercetin; basal diet + 0.2 g/kg vitamin E; and basal diet + the combination of quercetin (0.4 g/kg) and vitamin E (0.2 g/kg). The results show that the combination of quercetin and vitamin E significantly increased the serum alkaline phosphatase levels and the antioxidant status of the uterus (*p* < 0.05). In addition, the combination of quercetin and vitamin E significantly increased the concentrations of serum estrogen and progesterone, as well as elevated the expression of hypothalamic gonadotropin-releasing hormone-1 and follicular cytochrome P450 family 19 subfamily A member-1 (*p* < 0.05). We also found that the calcium levels of the serum and uterus were significantly increased by the synergistic effects of quercetin and vitamin E (*p* < 0.05), and they also increased the expression of Ca^2+^-ATPase and the mRNA expression of calcium-binding-related genes in the uterus (*p* < 0.05). These results are consistent with the increased eggshell quality of the laying hens under heat stress. Further, the combination of quercetin and vitamin E significantly increased the uterine morphological characteristics, such as the height of the uterine mucosal fold and the length of the uterine mucosa villus of the heat-stressed laying hens. These results collectively improve the uterine function, serum and uterine calcium concentration, eggshell strength, and eggshell thickness (*p* < 0.05) in heat-stressed laying hens. Taken together, we demonstrated in the present study that supplementing the combination of dietary quercetin and vitamin E alleviated the effects of heat stress and improved calcium metabolism, hormone synthesis, and uterine function in the heat-stressed laying hens. Thus, the supplementation of the combination of quercetin and vitamin E alleviates oxidative stress in the eggshell gland of heat-stressed laying hens, thereby promoting calcium concentration in the serum and eggshell gland, etc., in laying hens. Hence, the combination of quercetin and vitamin E promotes the reproductive performance of the laying hens under heat stress and can also be used as a potent anti-stressor in laying hens.

## 1. Introduction

Reports have indicated that laying hens for optimal performance are at a comfort zone at an ambient temperature between 18 °C and 23 °C [1,2]. However, characteristics of heat stress begin to manifest when the temperature goes beyond 30 °C [1,2,3]. Reproductive performance and immune function are highly affected by excessive heat stress in breeder laying hens [3,4,5].

Heat stress usually affects almost all animals, including chickens. However, chickens are more sensitive to heat stress because their capacity to dissipate body heat is low. Hence, in chickens, excessive ambient temperature negatively influences their physiological performance and immunity [3,6,7]. Studies have reported that, compared to earlier chicken strains, modern chicken genotypes produce more heat due to their excessive metabolic activity primarily because of selective breeding for rapid growth and higher productivity. This selection process has led to several physiological and metabolic changes in modern chickens because of factors such as an increased growth rate, enhanced feed conversion efficiency, larger muscle mass, and altered physiology. These factors collectively contribute to the increased heat production observed in modern chicken genotypes compared to their earlier counterparts [6,7,8,9]. The reproductive performance of chickens under heat stress conditions decreases significantly. Heat stress in hens causes low-quality eggshell formation and reduces steroidogenesis [4,10]. In addition, the reproductive failure in heat-stressed hens is generally attributed to uterine and ovarian dysfunction. Thus, uterine and ovarian dysfunction have a strong correlation with oxidative stress caused by heat stress [4,10,11].

In poultry, eggshell formation is dependent on several biological processes, such as assembling hydrogen carbonate (HCO), calcium, and the secretion of ions in the shell gland and lumen. In the uterus, the levels of H^+^ and phosphate decrease, thereby contributing to eggshell formation [12]. Thin eggshells are usually formed when absorption of calcium from the intestinal tract is inhibited, and also when there is an inhibition of calcium transport by the uterine mucosa [12]. This indicates that calcium is important for eggshell formation in laying hens [13]. In addition, estrogen plays essential functions in maintaining calcium homeostasis in laying hens [13]. However, environmental factors such as heat stress decrease the overall physiological function of animals [14]. Therefore, promoting estrogen synthesis is vital for promoting calcium accumulation, which can promote quality eggshell formation in laying hens. Dietary supplements can influence estrogen and calcium levels in the reproductive organs and serum of chickens [15]. Therefore, during oxidative stress, the dietary application of supplements such as antioxidants may improve the functions of the reproductive organs [15,16]; therefore, it is important to develop dietary targets to improve the eggshell quality of heat-stressed laying hens via increasing the concentrations of estrogen and calcium.

Studies have identified several dietary supplements, such as flavonoids, vitamins, etc., with the potential to alleviate the effects of heat stress in poultry [3,9].

Quercetin is an active flavonoid that possesses several biological properties, such as anti-aging, anti-inflammatory, antioxidant, and immunological properties [17]. Quercetin attenuates oxidative stress by alleviating the production of reactive oxygen species and protects cellular macromolecules, as well as inhibits lipid peroxidation [18,19].

Vitamin E is a strong antioxidant that is well known for its role in protecting the tissue integrity of lipid materials of an organism via alleviating spontaneous autoxidation [16,20]. Vitamin E is usually given to chickens in a bioavailable form as α-tocopherol [16]. Studies have indicated that vitamin E plays a scavenging role by regulating the balance between ROS (reactive oxygen species) production. It also exerts anti-stressor properties and promotes liver function in poultry [17,21,22,23]. Heat stress can increase liver and blood malonyldialdehyde concentrations to cause oxidative stress, and hence decrease egg production [24]. However, reports have indicated that vitamin E supplementation could significantly attenuate oxidative stress and inflammation induced by heat stress in chickens [23,24].

Several studies have reported the effects of many flavonoids, such as curcumin, salidroside, turmeric powder, etc., on the production performance and immunity of chickens. It was reported that flavonoids such as curcumin alleviate heat stress in quails via regulating the expression of heat shock protein 70 and hepatic nuclear transcription factors [3,25,26,27].

Studies on the effects of individual quercetin and vitamin E on the productive performance of poultry have been conducted; however, there are no studies on the impacts of the combination of dietary quercetin and vitamin E on the reproductive performance of heat-stressed laying hens. Owing to the previous literature, we hypothesized that combining these two potent antioxidants (quercetin and vitamin E) would alleviate the negative effects of heat stress on laying hens. Hence, the purpose of this present study was to determine the impacts of combining quercetin and vitamin E on the uterine function, calcium metabolism, and hormone synthesis in heat-stressed breeder hens.

## 2. Materials and Methods

### 2.1. Experimental Design

This study was approved by the Animal Care and Use Committee of Sichuan Agricultural University. Animals used in this experiment were cared for under the guidelines stated in the Guide for the Care and Use of Agricultural Animals in Agricultural Research and Teaching of Sichuan Province, China (Certification No. SYXK2019-187 (Chengdu, China)). The experimental chickens, Tianfu breeder hens (120-days-old), were obtained from the Sichuan Agricultural University Poultry Breeding Unit. The “Tianfu broiler chicken” is a fast-growing, high-quality jute-feathered green foot chicken developed by the Poultry Research Breeding Group of Sichuan Agricultural University and Sichuan Banghe Agricultural Science and Technology Co., Ltd., in Chengdu, China. Tianfu broiler breeder hens are a highly productive indigenous breed, available in both meat-type and egg-type varieties with rapid growth rates. The egg-type variety typically reaches peak egg production (>95%) at around 30–35 weeks of age, but this gradually declines to less than 60% after 60 weeks [17]. A sum of 400 laying hens (120-days-old) were randomly allotted into four (4) dietary treatments containing 100 birds each. Each treatment had 4 replicates consisting of 25 birds each. The four (4) groups of chickens were fed different diets, including (1) basal diet only; (2) basal diet supplemented with dietary quercetin (0.4 g/kg); (3) basal diet supplemented with dietary vitamin E (0.2 g/kg); and (4) basal diet supplemented with the combination of quercetin and vitamin E (0.4 g/kg and 0.2 g/kg) for an 8-week experimental period. Throughout this study, we set the photoperiod at 16 h of light and 8 h of darkness. The experiment commenced after a 10-day adaptation period. In addition, we measured the temperature and humidity in the chicken’s pen daily at exactly 9:00 am, 13:00 pm, 17:00 pm, and 21:00 pm and, also, we set the ambient temperature transformation at 34 ± 2 °C for 8 h/d (9:00 am–5:00 pm), which was followed by 22 °C to 28 °C for 16 h/d. Throughout the experiment, the relative humidity in the chicken’s pen was at 50 to 65%. The hens were provided with drinking water ad libitum. The composition and nutritional values of the basal diet used in this study have been previously reported [17]. The recommended levels of individual vitamin E and quercetin inclusion were chosen owing to the previous literature [17,19,28].

### 2.2. Sample Collection

At the end of the 8-week experimental period, 8 laying hens per group were randomly selected and weighed, and the blood samples were collected via the wing vein; thereafter, the hens were euthanized for tissue sample collection. The blood samples were centrifuged at 3000× *g* for 15 min at 4 °C and stored at −80 °C for subsequent biochemical analysis. After the euthanization, the hypothalamus, pituitary, small yellow follicles (SYF), and shell gland (uterus) were collected immediately. The samples of hypothalamus, pituitary, and SYF tissues were stored at −80 °C for subsequent qRT-PCR analysis. Sections of approximately 3 cm in length were cut off from the middle of the uterus and were instantly fixed in 4% paraformaldehyde for histological analyses. Part of the uterus was stored at −80 °C for the analysis of oxidative status, enzymatic activity, and gene expression.

### 2.3. Egg Quality

At the end of the 8-week experimental period, a total of 64 eggs (4 eggs per replicate; 16 eggs per treatment) were randomly selected and were subjected to an egg quality test. Egg quality indices such as egg weight were measured using an electronic measuring scale, whereas the eggshell strength was measured using an eggshell force gauge model II (Robotmotion Co., Ltd., Takanawa Minato-ku, Tokyo, Japan), and the shell thickness was measured by averaging three sections (air cell, equator, and sharp end) of each egg using a vernier caliper.

### 2.4. Determination of Serum Biochemical Parameters, Progesterone, and Estradiol Levels

The following biochemical parameters were determined from the serum: glucose (GLU), calcium, and alkaline phosphatase (ALP), using commercial kits following the manufacturer’s instructions (Nanjing Jiancheng Bioengineering Institute, Nanjing, China). The serum progesterone and estradiol levels were detected using enzyme-linked immunosorbent assay (ELISA) kits according to the manufacturer’s instructions (Baolai Biotechnology Co., Ltd., Yancheng, China).

### 2.5. Determination of Calcium Level and Action of Ca^2+^-ATPase

An amount of 0.2 g frozen shell gland (uterine) mucosae was homogenized in 2.0 mL of deionized water. After centrifugation for 20 min at 2500× *g* at 4 °C, the supernatant was collected to measure the calcium concentration and activity of Ca^2+^-ATPase. The protein content of the supernatant was measured with a BCA Protein Assay Kits (Nanjing Jiancheng Bioengineering Institute, Nanjing, China). Moreover, the calcium concentration in the serum and shell gland was measured by the commercial kits, whereas the activity of Ca^2+^-ATPase in the shell gland was detected using commercial kits according to the manufacturer’s instructions (Baolai Biotechnology Co., Ltd., Yancheng, China).

### 2.6. Morphological Analysis of the Uterus Heat-Stressed Hens

After fixing the shell gland in a 4% paraformaldehyde for 24 h, it was further subjected to morphological analysis. It was soaked via a graded series of ethanol and xylene, embedded in paraffin, and sectioned. Thereafter, the section was deparaffinized with xylene and rehydrated through a graded dilution of ethanol, and then stained with hematoxylin and eosin (HE). Thereafter, the images of the shell gland were acquired using an Olympus simon-01 microscope (Olympus Optical, Beijing, China). Furthermore, the uterine morphological characteristics such as the width (between 2 adjacent uterine mucosal folds) and the width of the uterine mucosal folds were measured using ImagePro Plus 6.0 software (Media Cybernetics). Three (3) samples were examined for each uterine tissue, with 3 images taken per sample.

### 2.7. Assessment of the Oxidative Status in the Shell Gland of Heat-Stressed Hens

The uterine tissue (0.2 g) was precisely weighed and homogenized in 2 mL of ice-cold PBS. After being centrifuged at 12,000× *g* for 10 min at 4 °C, the supernatant was collected to measure the antioxidant indices. The protein content of the supernatant was measured using a BCA Protein Assay Kit. Then, the following antioxidant indices—catalase (CAT) activity, glutathione peroxidase (GSH-Px) activity, superoxide dismutase (SOD) activity, and malondialdehyde (MDA) content—were assessed in the serum and shell gland using commercial biochemistry kits according to the manufacturer’s instructions (Nanjing Jiancheng Bioengineering Institute, Nanjing, China).

### 2.8. Total RNA Extraction and qPCR

Total RNA was extracted from the SYF, hypothalamus, pituitary, and shell gland (uterus) following the procedures described previously [29], using TRIzol reagent (Takara, Dalian, China), according to the manufacturer’s instructions. Then, the concentration and purity of the extracted RNA were determined using Nanodrop 2000C (Thermo Fisher Scientific, Waltham, MA, USA) with the absorbance ratio of A260/280. Thereafter, the PrimeScript RT Reagent Kit (Takara, Dalian, China) was used to synthesize the single-strand cDNA according to the manufacturer’s instructions. Then, the single-strand cDNA was used for qRT-PCR analysis through the CFX96 Real-Time System (Bio-Rad, Hercules, CA, USA) at suitable conditions: 95 °C for 3 min, 40 cycles of 95 °C for 10 s, and annealing temperature (Table 1) for 20 s, followed by a final extension at 72 °C for 20 s, with a melt curve analysis performed at 65~95 °C. The amplification efficiencies of the target genes ranged from 95% to 105%. Each qRT-PCR reaction was performed with volumes of 15 µL containing 6.25 µL TB Green TM Premix (Takara), 0.3 µL forward and reverse primers, 1.5 µL cDNA, and 6.65 µL DNase/RNase-Free Deionized Water (Tiangen, Beijing, China). *β-ACTIN* was used as the housekeeping gene for the genes determined in the SYF, hypothalamus, and pituitary tissues, whereas the hypoxanthine phosphoribosyltransferase 1 (*HPRT1*) was used as the housekeeping gene for the genes determined in the shell gland. *HPRT1* is suitable for the normalization of gene expression in the uterus of chickens. The relative gene expression levels were analyzed by the 2^−ΔΔCt^ method [30] after normalization against *β-ACTIN* or *HPRT1*. In the determination of gene expression, all samples were measured in triplicate and the experiment was performed twice.

### 2.9. Statistical Analysis

In this study, all the data were analyzed by one-way analysis of variance (ANOVA) using GraphPad Prism version 6.01 statistical package for Windows (GraphPad Software Inc., San Diego, CA, USA) and SPSS 20 Statistical Analysis Software (SPSS Inc., Chicago, IL, USA). Therefore, all the experimental data were presented as the mean ± standard deviation (SD), and differences among treatments were examined using Tukey’s test. Calculated Δ Ct (corrected sample) = mean value of target gene − mean value of internal reference gene; ΔΔ Ct = Δ Ct − mean value of the control group. The values were significantly different at *p* < 0.05.

## 3. Results

### 3.1. Effects of Quercetin (Q), Vitamin E (VE), and Combination of Quercetin and Vitamin E (Q + VE) on Production Performance of Heat-Stressed Hens

**Figure 1:**
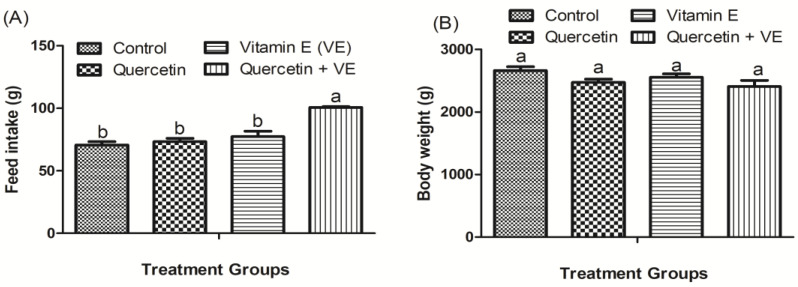
Impacts of quercetin (Q), vitamin E (VE), and combination of quercetin and vitamin E (Q + VE) on feed intake (g/day/laying hen) (**A**) and body weight (g) (**B**) of heat-stressed hens. The values are presented as the mean *±* SD. Bars without the same letter differed significantly (*p* < 0.05).

As shown in Figure 1, the results indicated that dietary vitamin E and the combination of quercetin and vitamin E significantly increased the feed intake compared to the control (*p* < 0.05) after the 8-week experimental period. However, there was no significant difference between the control, quercetin, and vitamin E groups (*p* > 0.05). This indicates that the supplemental diets were palatable for the chickens to consume. Interestingly, for the body weight, there were no significant differences observed among all four dietary groups (*p* > 0.05) after the 8-week experimental period.

**Figure 2:**
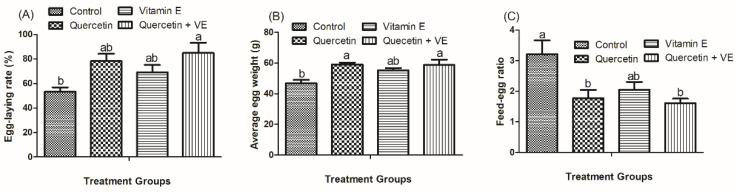
Effects of supplementation with quercetin (Q), vitamin E (VE), and combination of quercetin and vitamin E (Q + VE) on egg-laying rate (**A**), average egg weight (**B**), and feed–egg ratio (**C**) throughout the 8-week experimental period. The values are presented as the mean *±* SD. Bars without the same letter differed significantly (*p* < 0.05). Feed–egg ratio = daily feed consumption/average egg weight.

As shown in Figure 2, we recorded that, compared to the control group, the supplementation of Q + VE improved the egg-laying performance and average egg weight, as well as the feed–egg ratio (*p* < 0.05), after the 8-week feeding period. However, there was no difference in the levels of egg-laying performance, average egg weight, and the feed-egg ratio in the individual quercetin and vitamin E groups and the combination group (*p* < 0.05). This indicates that chickens in the combination group recorded higher feed efficiency.

### 3.2. Effects of Dietary Quercetin (Q), Vitamin E (VE), and Combination of Quercetin and Vitamin E (Q + VE) on Eggshell Quality

**Figure 3:**
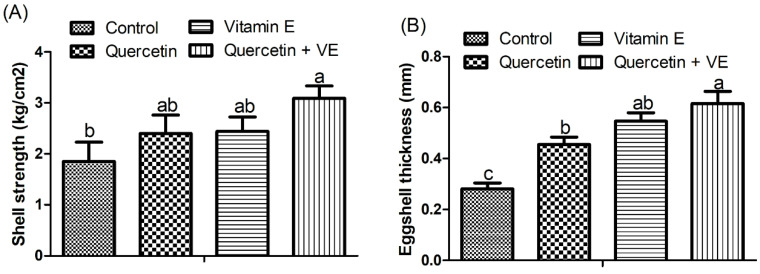
Effects of dietary quercetin (Q), vitamin E (VE), and combination of quercetin and vitamin E (Q + VE) on eggshell strength (**A**) and shell thickness (**B**). The values are presented as the mean *±* SD. Bars without the same letter differed significantly (*p* < 0.05).

As shown in Figure 3, in comparison with the control group, the supplementation of Q + VE notably enhanced the eggshell strength and eggshell thickness (*p* < 0.05) compared with the control group. Importantly, the individual quercetin and vitamin E also significantly improved the eggshell thickness compared to the control group (*p* < 0.05).

### 3.3. Effects of Dietary Quercetin (Q), Vitamin E (VE), and Combination of Quercetin and Vitamin E (Q + VE) on Serum Biochemical Parameters, Progesterone and Estradiol Levels

In Table 2, we observed that the biochemical indices such as glucose and alkaline phosphatase were significantly influenced by the dietary combination of quercetin and vitamin E compared to the control (*p* < 0.05). Interestingly, the supplementation of the individual quercetin and vitamin E into the diet of laying hens significantly decreased the serum ALP activity compared with the control group (*p* < 0.05).

**Figure 4:**
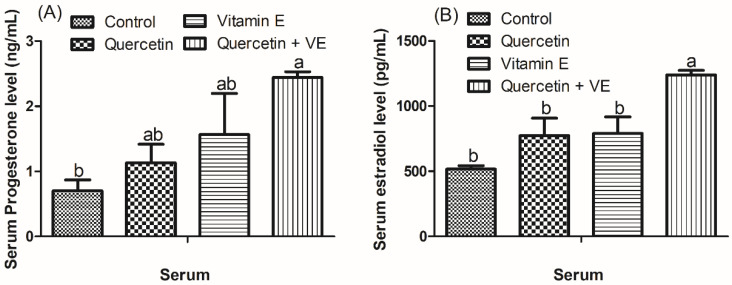
Effects of dietary supplementation with quercetin (Q), vitamin E (VE), and combination of quercetin and vitamin E (Q + VE) on the serum progesterone (**A**) and estradiol (**B**) levels of the heat-stressed hens. The values are presented as the mean *±* SD. Bars without the same letter differed significantly (*p* < 0.05).

Moreover, in Figure 4, the supplementation of Q + VE significantly increased the serum estradiol and progesterone levels compared with the control group (*p* < 0.05) after the 8-week feeding period. Further, supplementation of quercetin and vitamin E alone significantly increased the serum estradiol concentration compared with the control group (*p* < 0.05).

### 3.4. Effects of Dietary Quercetin (Q), Vitamin E (VE), and Combination of Quercetin and Vitamin E (Q + VE) on Calcium Concentration and Ca^2+^-ATPase Activity

**Figure 5:**
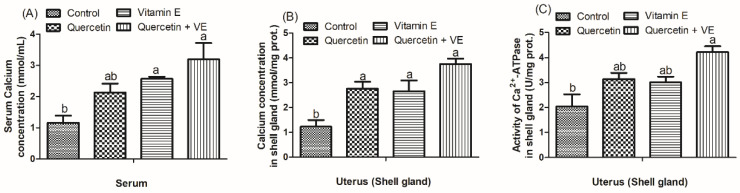
Effects of dietary supplementation with quercetin (Q), vitamin E (VE), and combination of quercetin and vitamin E (Q + VE) on the serum calcium concentration (**A**), uterine calcium concentration (**B**), and activity of Ca^2+^-ATPase in the shell gland (**C**). The values are presented as the mean *±* SD. Bars without the same letter differed significantly (*p* < 0.05).

Figure 5 shows the calcium concentration in both the serum and shell gland. The results indicate that Q + VE significantly elevated the levels of calcium concentration in both the serum and the shell gland compared to the control group, as well as increased the activity of Ca^2+^-ATPase in the shell gland of the heat-stressed hens (*p* < 0.05). Furthermore, it was observed that vitamin E alone also significantly increased the serum calcium levels, and both quercetin and vitamin E significantly increased the calcium level in the shell gland compared with the control group (*p* < 0.05).

### 3.5. Effects of Dietary Quercetin (Q), Vitamin E (VE), and Combination of Quercetin and Vitamin E (Q + VE) on Oxidative Status of the Uterus and Serum

Table 3 summarizes the effects of dietary supplementation with Q + VE on the oxidative status of the uterus (shell gland) and serum. The results show that Q + VE significantly increased the levels of SOD, TAOC, CAT, and GSH-Px in the shell gland and serum of the heat-stressed hens (*p* < 0.05) after the 8-week feeding period. In addition, Q + VE supplementation significantly reduced the MDA content in all the tissues evaluated in this study (*p* < 0.05) compared with the control group. Furthermore, we have observed that the individual quercetin also increased the concentration of SOD in the serum, whereas both the individual quercetin and vitamin E increased the concentration of SOD in the uterus of the laying hens. In addition, supplementing the individual quercetin and vitamin E increased the levels of TAOC, GSH-Px, and CAT in the serum and uterus of the laying hens (*p* < 0.05); however, they both individually (quercetin and vitamin E) decreased the concentration of MDA in the serum and eggshell gland of the chickens (*p* < 0.05).

### 3.6. Effects of Dietary Quercetin (Q), Vitamin E (VE), and Combination of Quercetin and Vitamin E (Q + VE) on Gene Expressions in the Uterus of Heat-Stressed Hens

**Figure 6:**
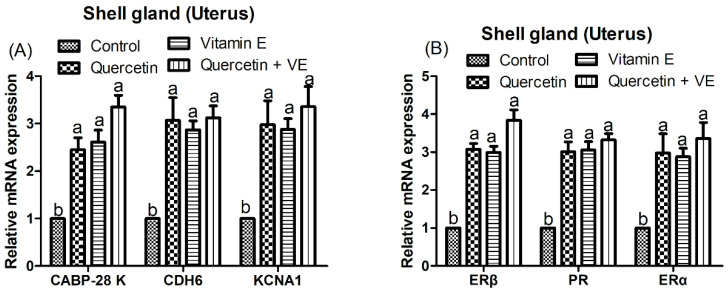
Effects of dietary supplementation with quercetin (Q), vitamin E (VE), and combination of quercetin and vitamin E (Q + VE) on the expression of genes such as calbindin 1 (*CABP-28 K*), cadherin 6 (*CDH6*), potassium voltage-gated channel subfamily A member 1 (*KCNA1*) (**A**) and estrogen receptors alpha (*ERα*) and beta (*ERβ*), and progesterone receptor (*PR*) (**B**) in the shell gland of heat-stressed hens. Different superscript letters indicate significant differences among the means (*p* < 0.05).

As shown in Figure 6, the dietary supplementation with the individual quercetin and vitamin E and the combination of quercetin and vitamin E significantly up-regulated the mRNA expressions of *ERα*, *ERβ*, *calbindin 1* (*CABP-28 K*), *cadherin 6* (*CDH6*), and potassium voltage-gated channel subfamily A member 1 (*KCNA1*) in the shell gland of the laying breeder hens compared with the control group (*p* < 0.05) (Figure 6). In addition, Q, VE, and Q + VE exerted synergistic effects on the mRNA expression of the androgen receptor (*AR*) and progesterone receptor (*PR*) in the shell gland (*p* < 0.05) of the heat-stressed laying hens.

### 3.7. Effects of Dietary Quercetin (Q), Vitamin E (VE), and Combination of Quercetin and Vitamin E (Q + VE) on Gene Expressions in the SYF, Hypothalamus, and Pituitary of Heat-Stressed Hens

**Figure 7:**
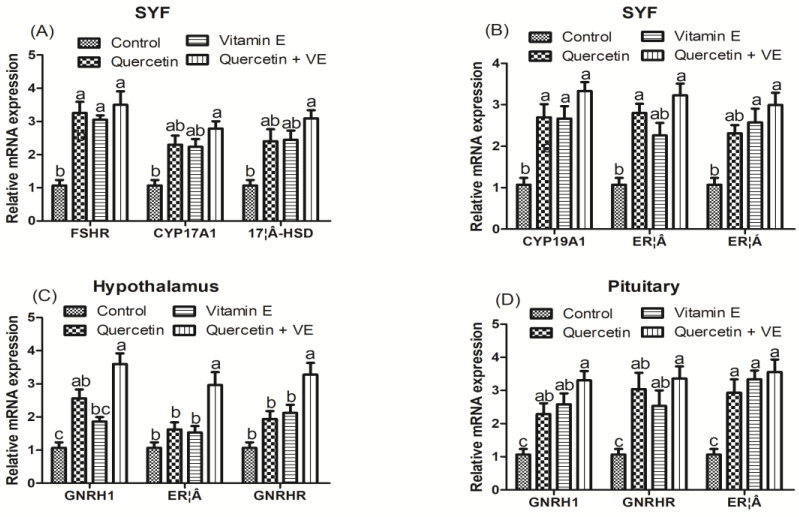
Effects of dietary supplementation with quercetin (Q), vitamin E (VE), and combination of quercetin and vitamin E (Q + VE) on gene expression in the small yellow follicles (SYFs) (**A**,**B**), hypothalamus (**C**), and pituitary (**D**) of heat-stressed hens. Different superscript letters indicate significant differences among the means (*p* < 0.05).

Figure 7 represents the data on the mRNA expressions of steroidogenic genes in the SYF (Figure 7A,B). The results show that dietary Q + VE as well as the individual quercetin and vitamin E remarkably increased the expression of the follicle-stimulating hormone receptor (*FSHR*), cytochrome P450 family 17 sub-family A member 1 (*CYP17A1*), hydroxysteroid 17-beta dehydrogenase (*17β-HSD*), cytochrome P450 family 19 subfamily A member 1 (*CYP19A1*), estrogen receptor 2 (*ERβ*), and estrogen receptor 1 (*ERα*) compared with the control group (*p* < 0.05). Moreover, the mRNA expressions of the hypothalamic gonadotropin-releasing hormone 1 (*GNRH1*), estrogen receptor 2 (*ERβ*), and hypophyseal gonadotropin-releasing hormone receptor (*GNRHR*) were up-regulated in the hypothalamus and pituitary by dietary supplementation with Q + VE (Figure 7C,D; *p* < 0.05).

### 3.8. Effects of Dietary Quercetin (Q), Vitamin E (VE), and Combination of Quercetin and Vitamin E (Q + VE) on the Morphological Analysis of the Uterus

**Figure 8:**
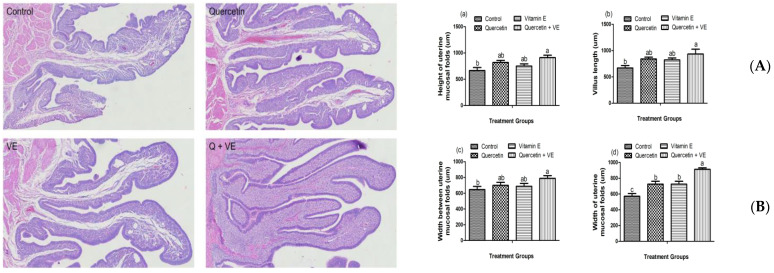
Effect of quercetin (Q), vitamin E (VE), and combination of quercetin and vitamin E (Q + VE) on the morphological changes of the shell gland (uterus). (**A**) Morphology of the uterus. Scale bar = 20 μm. (**B**) Morphological characteristics of the uterus. (**a**) The height of the uterine mucosal fold (mm). (**b**) The length of villus in the endometrium (mm). (**c**) The width of the 2 adjacent uterine mucosal folds (mm). (**d**) The width of the uterine mucosal folds (mm). Different superscript letters indicate significant differences among the means (*p* < 0.05).

Figure 8A shows the morphological characteristics of the uterine tissues among the treatment groups. The results show that the height of the uterine mucosal fold was significantly increased by the dietary combination of quercetin and vitamin E and individual quercetin and vitamin E supplementation (Figure 8(Aa); *p* < 0.05). Furthermore, we found that supplementation of individual dietary quercetin and vitamin E, as well as the combination of quercetin and vitamin E, significantly increased the length of the uterine mucosa villus in the uterine mucosal (Figure 8(Ab); *p* < 0.05), as well as increased the width between two adjacent uterine mucosal folds and the width of the uterine mucosal folds (Figure 8(Bc,Bd); *p* < 0.05).

## 4. Discussion

Oxidative stress is a major factor that causes a sharp decline in the performance of hens due to its organ damage sensitivity [31,32]. In this study, it was revealed for the first time that supplementing the combination of dietary quercetin and vitamin E exerts synergistic effects on the mechanisms that promote eggshell quality and uterine functions in heat-stressed hens. It was revealed in this study that alkaline phosphatase (ALP) was significantly increased by the dietary Q + VE. ALP facilitates tissues mineralization to form hard tissues, thereby promoting eggshell mineralization. Thus, ALP is fundamentally linked to bone metabolism in chickens. It is a marker of bone formation, eggshell formation, and health, playing a vital role in the mineralization and overall integrity of the skeletal system. Therefore, monitoring ALP levels can provide valuable insights into the growth, development, and bone health of chickens [33]. The estradiol level in stressed chickens usually reduces remarkably compared to hens in a comfortable environment [3,34]. Studies have reported that heat stress increases the level of serum hormone cortisol (COR), thereby decreasing the expression of estradiol (E2), luitenizing hormones (LHs), and follicle-stimulating hormones (FSHs) in laying hens [34]. In the present study, supplementing the combination of quercetin and vitamin E significantly increased the levels of reproductive hormones in heat-stressed laying hens. It was also reported that oxidative stress in reproductive organs induced by heat stress causes a significant decrease in reproduction due to excessive inflammation and organ damage [3,35]. In this present study, we observed that the antioxidant capacity of the hens in the Q + VE group was enhanced, and this indicates that the synergistic effects of Q + VE could reduce uterine oxidative stress in the heat-stressed hens. Chemical metabolites obtained from the hydrolysis of quercetin exert their biological function on reproductive organs [35,36,37]. Estrogen promotes the steroidogenesis and antioxidant capacity of the reproductive organs [38]. It was revealed in this present study that the interactive effects of dietary Q + VE up-regulated the mRNA expression of steroidogenic genes. *CYP19A1* plays an important role in converting androgens to estrogens [39]. Therefore, the elevation of *CYP19A1* induced by the combination of Q + VE could be responsible for the increased serum estradiol level observed in this study. Consistently, the elevated mRNA expression of estrogen receptors in the hypothalamus, SYF, and shell gland also supported an increased estrogen level in the Q + VE group. Moreover, we found that the expression of *CYP17A1* and *17β-HSD* (mainly located in the theca cells) that mediates androgen synthesis [40] was elevated by the supplementation of Q + VE. However, further validation is required to ascertain the specific mechanism through which Q + VE exerts effects on theca cells. Studies have shown that a decrease in the function of the hypothalamic–pituitary–gonadal axis causes reproductive failure in chickens [41]. Therefore, the up-regulation of the mRNA expression of the hypothalamic *GNRH1* (gonadotropin-releasing hormone) and hypophyseal GNRHR (gonadotropin-releasing hormone receptor) observed in the present study suggests that the supplementation of Q + VE promoted the secretion of gonadotropin-releasing hormones in the heat-stressed hens. Follicle-stimulating hormones promote follicular development [42]. Therefore, the elevated expression of *FSHR*s obtained in this present study shows that follicular development may be improved by Q + VE. Therefore, this present study revealed that the supplementation of Q + VE increased the estrogen synthesis via promoting the transcription of follicular *CYP19A1* and hypothalamic *GNRH1*.

Furthermore, in the control group, we observed a damaged morphology of the shell gland, which was characterized by a messy distribution of tubular gland cells. The tubular gland cells in the shell gland are responsible for secreting calcium ions and pigment, which, in turn, promote the formation of the eggshell [36,37]. During oxidative stress, the tubular gland cells become dysfunctional, and the overproduction of eggs may reduce the glandular density. Eggshell formation mainly occurs in the isthmus and uterine parts of the oviduct [43,44]. We observed in this present study that the synergy of dietary quercetin and vitamin E significantly increased the height of the uterine mucosal fold and length of the uterine mucosa villus in the uterine mucosal of the heat-stressed laying hens. This reveals that the dietary combination of quercetin and vitamin E enhanced the secretory ability of the uterine mucosa, which promotes the transport of ions into the uterus fluid, to increase calcification, leading to the improvement in the eggshell quality of heat-stressed laying hens. This is consistent with a previous study that reported that the supplementation of N-carbamylglutamate improves eggshell quality by promoting the uterine characteristics of laying hens [44].

In addition, dietary supplementation of Q + VE significantly increased the expression of antioxidant parameters such as CAT and GSH-Px in the shell gland of the heat-stressed chickens. This indicates that the synergy of quercetin and vitamin E has the ability to attenuate free radicals in the uterus of heat-stressed hens. A previous study explained that alleviating oxidative stress using dietary supplements could reduce apoptosis and tissue damage [45]. This shows that the morphological characteristics observed in the uterus of the heat-stressed laying hens indicated that the synergistic effects of quercetin and vitamin E alleviate oxidative stress and tissue damage due to their antioxidant properties. Studies have reported that exposing chickens to high temperatures could decrease serum protein and calcium levels, which are needed for egg formation [46,47]. Moreover, calcium has been reported to regulate the transcription of aromatase in the theca cells of hens [48]. It was shown that estrogen concentration is positively correlated with calcium concentration in hens [49,50]. Thus, a high egg-laying performance in hens is accompanied by an increased serum calcium level [51]. Hence, consistent with the above previous studies, this present study showed that the dietary supplementation of the Q + VE group increased the serum estrogen and calcium concentrations. Studies have indicated that estradiol regulates calcium metabolism and shell calcification in poultry [52]. Moreover, a study also increased the eggshell thickness by injecting estradiol in laying hens, and, also, eggshell thickness is correlated with eggshell strength [53]. Reports have indicated that feed intake significantly influence eggshell quality in laying hens because it enables free available serum calcium to combine with plasma protein and/or other substances to ensure adequate blood Ca^2+^, which can actively help in eggshell formation [3]. Therefore, it was revealed in this present study that the combination of quercetin and vitamin E increased the levels of serum estrogen, which subsequently promoted eggshell quality in the heat-stressed laying hens. In addition, it was observed in this study that the combination of quercetin and vitamin E increased the calcium concentration in both the serum and uterus; this indicates that calcium was adequately absorbed and efficiently utilized, which further promotes eggshell strength and calcification in the Q + VE group.

Therefore, the results obtained in this study reveal that the supplementation of Q + VE improved uterine function and eggshell quality, which were associated with increased estrogen and calcium levels. Ca^2+^-ATPase and CaBP-28k are the two major components involved in calcium transportation in the uterus, and they are both localized in the tubular gland cells [52]. In this present study, we found a consistent increase in the expression of Ca^2+^-ATPase and CABP-28 K, which was consistent with the increased glandular density in the Q + VE group. In addition, calcium-binding-related genes such as *THBS2*, *CDH6,* and *KCNA1* were increased. This indicates that Q + VE modulates calcium transportation and signalling in the heat-stressed laying hens.

## 5. Conclusions

Taken together, we demonstrated in the present study that supplementing the combination of dietary quercetin and vitamin E in heat-stressed laying hens improved the eggshell quality via increasing the estrogen synthesis, calcium level, and antioxidant capacity of the uterus. Hence, this promotes the reproductive performance of laying hens under heat stress. The results obtained from the present study show that the combination of quercetin and vitamin E can alleviate the effects of heat stress in laying hens. Therefore, this study provides information on the potential anti-stressor function of Q + VE supplementation, which may be useful in developing a new improved strategy for promoting uterine functions, eggshell quality, and reproductive functions in heat-stressed hens.

## Figures and Tables

**Table 1:** Primers used for quantitative real-time polymerase chain reaction (qRT-PCR).

Gene	Sequence (5′–3′)	Product Length (bp)	Annealing Temperature (°C)
*FSHR*	F: GAGGAGGTGAAGAAGATGCGGATG R: TGAGCCACTCTGTTGCCATACC	105	60
*CYP17A1*	F: CTTCAGGTGTTTCTCTTCCTCCTC R: CTGTGGTTTCATGGCTGGATC	131	59.82
*CYP19A1*	F: TCATCGCCTCCATCGTCTAC R: TCTTACTGCGCGTCTTCTGG	240	57.79
*17* *β* *-HSD*	F: CCCTCACCCAGCCCGACTTC R: GCCGTTGGTGGAGGTGTTACAG	179	58
*ER* *β*	F: GGCAAGCAGCACGGTGGAC R: CTTCTGCCACTCCTCCCTTTGC	129	59
*ER* *α*	F: ACGGCACCAACGAGGAGATCC R: CTTCCCGTTCACCTGGCACTTC	175	60.67
*GNRHR*	F: TGTGCTGTGTGCAACGACTA R: CAGGCCTGGCAACTCTTTCT	167	57
*GNRH1*	F: CTGCAGGACGAGATGTGCAA R: AGGTCTGAAAGGCGAACAGG	175	60.67
*CABP-28 K*	F: ACATCCAGGGAGAGGTTTCCT R: GTGGGACATGGTGCCTTGAG	208	60.20
*CDH6*	F: ATCGTCGCCTTCTTCGAGTT R: ATCCCATCCTCCGTTGTCCT	150	59
*PR*	F: GTGATGGCATGGGACATAGCTC R: TGGCGTAGACCTTGCGGATAA	90	58
*HPRT1*	F: GTGATGGCATGGGACATAGCTC R: TGGCGTAGACCTTGCGGATAA	90	58
*β* *-actin*	F: ATCCGGACCCTCCATTGTC R: AGCCATGCCAATCTCGTCTT	120	60

**Table 2:** Effects of dietary supplementation with quercetin (Q), vitamin E (VE), and combination of quercetin and vitamin E (Q + VE) on serum biochemical parameters of heat-stressed hens.

Item ^1^	Control	Quercetin	Vitamin E	Q + VE
GLU (mmol/L)	16.15 ± 2.09 ^a^	14.51 ± 2.68 ^ab^	13.15 ± 1.79 ^bc^	11.99 ± 1.76 ^c^
ALP (U/L)	295.93 ± 65.02 ^a^	234.09 ± 66.30 ^b^	179.15 ± 79.94 ^bc^	132.99 ± 42.57 ^c^

^a–c^ mean ± standard deviation values within the same row sharing a common superscript letter are not statistically different at *p* < 0.05. ^1^ GLU, glucose; ALP, alkaline phosphatase. Q + VE = combination of quercetin and vitamin E (Q + VE).

**Table 3:** Effects of dietary quercetin (Q), vitamin E (VE), and combination of quercetin and vitamin E (Q + VE) on antioxidant enzymes and MDA levels in the serum and uterus of heat-stressed hens.

Parameters ^1^		Treatments			
	Tissue	Control	Q	VE	Q + VE
SOD	Serum	17.53 ± 3.09 ^c^	20.48 ± 12.14 ^b^	16.77 ± 2.53 ^c^	33.53 ± 10.07 ^a^
	Uterus	24.20 ± 5.61 ^c^	32.23 ± 7.61 ^ab^	30.14 ± 7.14 ^ab^	35.01 ± 6.22 ^a^
TAOC	Serum	14.81 ± 4.13 ^b^	20.18 ± 6.18 ^a^	19.70 ± 4.76 ^a^	24.27 ± 6.19 ^a^
	Uterus	11.58 ± 2.49 ^c^	19.21 ± 6.28 ^ab^	19.49 ± 6.12 ^ab^	22.08 ± 11.27 ^a^
GSH-Px	Serum	29.87 ± 7.58 ^c^	39.60 ± 5.13 ^b^	36.86 ± 6.35 ^b^	47.73 ± 6.81 ^a^
	Uterus	18.78 ± 3.65 ^c^	23.83 ± 7.68 ^b^	24.63 ± 5.23 ^ab^	29.21 ± 4.20 ^a^
CAT	Serum	16.12 ± 3.64 ^c^	22.01 ± 4.99 ^b^	22.68 ± 8.05 ^b^	29.20 ± 8.11 ^a^
	Uterus	29.25 ± 10.62 ^c^	43.96 ± 3.66 ^b^	49.49 ± 5.40 ^ab^	54.34 ± 5.59 ^a^
MDA	Serum	1.80 ± 0.59 ^a^	1.23 ± 0.52 ^b^	1.11 ± 0.53 ^c^	1.20 ± 0.64 ^b^
	Uterus	1.86 ± 0.53 ^a^	0.99 ± 0.61 ^b^	0.91 ± 0.43 ^b^	0.57 ± 0.17 ^c^

^a–c^ mean ± standard deviation values within the same row sharing a common superscript letter are not statistically different at *p* < 0.05. ^1^ Superoxide dismutase (SOD), total antioxidant capacity (T-AOC), glutathione peroxidase (GSH-Px), catalase (CAT), and methane dicarboxylic aldehyde (MDA).

## Data Availability

Data are contained within the article.
